# Long-term application of FYM and fertilizer N improve soil fertility and enzyme activity in 51^st^ wheat cycle under pearl millet-wheat

**DOI:** 10.1038/s41598-024-72076-w

**Published:** 2024-09-17

**Authors:** Sunita Sheoran, Dhram Prakash, Parmod Kumar Yadav, Rajeev Kumar Gupta, Nadhir Al-Ansari, Salah El-Hendawy, Mohamed A. Mattar

**Affiliations:** 1 Chaudhary Charan Singh Haryana Agricultural University, Hisar, Haryana 125004 India; 2 Department of Agronomy, Lovely Professional University, Jalandhar, Punjab 144001 India; 3 Department of Soil Science, Punjab Agricultural University, Ludhiana, Punjab 141004 India; 4 Department of Civil, Environmental, and Natural Resources Engineering, Lulea University of Technology, 97187 Luleå, Sweden; 5 Department of Plant Production, College of Food and Agricultural Sciences, King Saud University, P.O. Box 2460, Riyadh, 11451 Saudi Arabia; 6 Department of Agricultural Engineering, College of Food and Agricultural Sciences, King Saud University, P.O. Box 2460, Riyadh, 11451 Saudi Arabia

**Keywords:** Long-term, Season, Farmyard manure, Wheat growth stage, Soil organic carbon, Available nutrient, Enzyme activity, Agroecology, Agroecology

## Abstract

Our study from an ongoing research experiment initiated in *Rabi* 1967 at the Research Farm of CCS Haryana Agricultural University, Haryana, India, reports that during the 51st wheat cycle in pearl millet-wheat sequence, adding FYM in both seasons significantly impacted various soil parameters at different wheat growth stages compared to the *rabi* season. The application of 15 t of FYM ha^−1^ resulted in a considerable increase in dissolved organic carbon content (9.1–11.2%), available P (9.7–12.1%), and available S (12.6–17.1%), DHA levels by 7.3–22.0%, urease activity (10.1 and 17.0%), β-Glucosidase activity (6.2–8.4%), and APA activity (5.2–10.6%), compared to 10 t FYM ha^−1^. Application of N_120_ exhibited a considerable improvement in DHA (11.0–23.2%), β-Glucosidase (9.4–19.2%), urease (13.3–28.3%), and APA (3.3–6.2%) activity compared to control (N_0_). At stage 3, the box plot revealed that 50% of the available N, P, and S values varied from 223.1 to 287.9 kg ha^−1^, 53.0 to 98.2 kg ha^−1^, and 50.0 to 97.6 kg ha^−1^, respectively. Principal component analysis, with PC1 explaining 94.7% and PC2 explaining 3.15% of the overall variability, and SOC had a polynomial relationship with soil characteristics (R^2^ = 0.89 to 0.99). Applying FYM_15_ × N_120_ treatment during both seasons proved beneficial in sustaining the health of sandy loam soil in North-West India.

## Introduction

Soil is a crucial element of natural resources as it significantly impacts crop productivity and regulates nutrient dynamics in various agroecosystems. It also plays a vital role in governing a country's economics and food security^1^. The degradation of soil health is occurring due to the implementation of non-discerning nutrient management strategies in intensive agriculture, which is an unfavourable indication for the sustainability of the agricultural system^2^. Wheat is a highly cultivated cereal crop, and its versatility enables it to thrive in various soil and climate conditions^3^. India is the second largest global wheat producer, following China, with an area of 30.6 million hectares, production of 98.38 million metric tons, and productivity of 3216 kg per hectare^4^. As the amount of land available for each person to cultivate decreases, expanding the area under wheat is difficult. Therefore, the main focus should be on enhancing productivity by applying the nutrients carefully and maintaining the health of the soil^5^. Optimizing nutrient management approaches and agronomic techniques and effectively managing microbiological activity can increase agricultural productivity^6,7^.

Long-term field experiments are essential tools to understand and evaluate the complex processes in the field as they provide sufficient time for crops, crop rotation, fertilizers, and manures to have a measurable impact on soil properties, fertility and productivity, and predict future changes^8–10^. Inorganic fertilizers directly or indirectly affect the soil's chemical, physical, and biological properties^11,12^. Adding 100% NPK fertilizers combined with FYM led to the highest levels of soil organic carbon (SOC), available N, P and K, and increased activity of various enzymes in vertisol soil under a soybean-wheat cropping system^13^. In an INM experiment conducted over 28 years, applying a combination of 50% N from FYM and 50% N from RDF during the *kharif* season (maize crop), and applying 100% RDF during *rabi* season (wheat crop) resulted in the highest soil available N content, measuring 375.2 kg N ha^−1^^14^. Applying 100% NPK + FYM to wheat at the tillering stage resulted in the soil's highest availability of Olsen P (13.7 mg kg^−1^)^15^. In another long-term study, the highest alkaline phosphatase activity (243.7 µg PNP g^−1^ h^−1^) was reported at the tillering stage of wheat with 100% NPK fertilizer combined with FYM^16^. In a long-term experiment under a wheat–maize cropping system, soil urease activity was higher with organic compost application than inorganic fertilizers^17^. In a long-term experiment on wheat–soybean crop rotation, applying 200 kg N ha^−1^ resulted in the highest activity of β-glucosidase^18^. Rani et al.^19^ recorded the changes in pH, EC, SOC, and available NPK content in the soil across several stages of pearl millet growth under the pearl-millet wheat cropping system, and INM showed the highest urease activity at heading and maturity stages. The highest DHA, urease, and β-glucosidase activities in a long-term experiment were recorded in NPK + FYM treatment^20^. In another study, long-term application of organic manure resulted in significant improvements in SOC, availability of N, P, K, and S, dehydrogenase, urease, and APA activity compared to INM and control treatments at different growth stages of maize^21^.

Manure enhances soil's water and nutrient availability by increasing SOC through direct C inputs or indirectly through the accumulation of belowground biomass, leading to increased agricultural productivity^22^. Soil microbial biomass and enzyme activity are more responsive to changes in soil management than total soil organic matter (SOM). Therefore, their assessment is a highly responsive indicator of SOM decomposition^23^. Various soil parameters regulate enzyme activity, including physicochemical properties, N supply and its dynamics, soil microbial community, vegetation type, crop growth stages and ecological disturbances^1^. Microbial activity tends to be more intensive during the intense growth stage of crops, particularly at flowering. However, it gradually decreases when the crop approaches maturity^24^. The temporal fluctuations in enzyme activities during various crop growth stages are crucial for comprehending the soil's nutrient-supplying capability and the crop's demands. Cereals are N-demanding crops, and N fertilizers significantly influence crops' quality and quantity^25^. However, combining fertilizers and organic manures has proven to be a highly advantageous strategy for achieving desired crop yields and improving soil quality^10^. It can also mitigate the adverse effects of intense farming and uneven fertilizer use^26^. Furthermore, it enhances dehydrogenase activity and the soil's bacteria, fungi, and actinomycetes population. Paying close attention to nutrient management is crucial for enhancing SOC levels, promoting agricultural output, and preserving ecosystems' health. Using organic manures is necessary for rehabilitating soil in semi-arid eco-regions, where the loss of SOM is greater and biological quality is low^27^.

As documented in the literature, extensive research has been conducted in India and worldwide regarding the effects of long-term fertilization and manuring on soil qualities at various stages of crop growth. More understanding is needed regarding the relationship between different soil parameters and their changes during various phases of crop growth. We hypothesize that adopting a combined approach involving organic manures and fertilizers during various seasons is the most rational method for preserving soil fertility and ensuring long-term soil health^22^. The main objective of this study was to investigate how the application of FYM during different seasons, along with varying levels of FYM and N fertilizer, affect the soil properties and their relationship with soil enzyme activity (DHA, β-Glucosidase, urease, and APA) at different stages of wheat growth in sandy loam soil of semi-arid region of North-West India.

## Materials and methods

### Study area

The region experiences a semi-arid climate characterized by an average annual rainfall of 443 mm and an average annual temperature of 24.8 °C. Approximately 90% of precipitation is obtained from the Southwest monsoon from May to October. The mean maximum temperature reached its lowest point in January and the highest in May. The climatic conditions during the present experimentation period are given in Fig. S1.

### Experimental design

The present study was conducted at Research Farm, Department of Soil Science, CCSHAU, Hisar (India), using an ongoing long-term field experiment established in October 1967. The experiment focuses on compensating fertilizer nitrogen through farmyard manure (FYM) under a pearl millet-wheat cropping system. The experimental site is situated at a latitude of 29° 16′ N and a longitude of 75° 75′ E in the North-Western region of India. The soil type was sandy loam, classified explicitly as Typic Ustochrept. The physicochemical properties of surface soil (0–15 cm) were analyzed at the beginning of the experiment in 1967. The measurements obtained were as follows: soil pH of 8.20, soil organic carbon (SOC) content of 0.47%, calcium carbonate (CaCO_3_) content of 1.10%, available nitrogen (N) content of 200 kg ha^−1^, available phosphorus (P) content of 26.0 kg ha^−1^, and available potassium (K) content of 498 kg ha^−1^^28^. The experiment used a split-plot design, with 10 m × 5 m subplots and three replications. The treatments in this study involved three different seasons of applying FYM: applying it to *rabi* crop (winter season), *kharif* crop (summer season), and applying it to *rabi* and *kharif* crops (both seasons). These treatments were conducted in the main plots. Additionally, three different levels of FYM were applied to each crop: 15, 30, and 45 t ha^−1^ year^−1^ until 2007–2008, and 5 (FYM_5_), 10 (FYM_10_), and 15 (FYM_15_) t ha^−1^ year^−1^ from 2008 to 2009 onwards, based on dry weight. One plot of FYM control (FYM_0_) was also assigned to each main plot. The main plot was subdivided into two subplots, one receiving no nitrogen (N_0_: 0 kg N ha^−1^) and another receiving 120 kg N ha^−1^ (N_120_) through urea application. Before sowing the crops, the FYM was added to the plough layer (15 cm). The FYM utilized in the experiment was analysed annually. The average nutrient composition of FYM applied during 2017–2018 was as follows: C (38.10%), N (0.85%), P (0.97%), K (1.87%), S (0.36%), Na (0.68%) and Ca (1.25%). Nitrogen was divided into two splits, half applied at the time of sowing and the remaining half after 25–30 days, for both crops under their respective treatments. From the beginning of the trial, only urea has been used as chemical fertilizer in all treatments. Following field preparation, wheat variety WH 1105 was promptly sowed following university recommendations. The wheat crop was harvested manually during the third week of April 2018. The crop was watered using canal water, and an irrigation of approximately 7.5 cm was applied as needed based on a visual assessment of the field. The crops were harvested around 2–5 cm above ground level, and the roots and stubble were then incorporated into the field.

### Soil sampling and analysis

Collecting, preparing, and analyzing soil samples involved obtaining two sets of soil samples from each plot at various wheat growth phases, spanning from the fourth week of October 2017 to the third week of April 2018. Soil samples were collected in triplicate from the surface layer (0–15 cm) at five different stages: Stage 1 (at sowing), Stage 2 (tillering), stage 3 (heading), stage 4 (maturity), and stage 5 (after harvesting). A single set of soil samples was air-dried, ground, passed through a 2 mm sieve, and stored in a plastic bag in ambient conditions for physicochemical analysis. Another set of moist soil samples was placed in a freezer at a temperature of 4 °C to be analyzed for enzyme activity.

#### Physicochemical properties

Sieved soil samples were tested for soil pH using a glass electrode and potentiometric method^29^ and electrical conductivity (EC) by adopting the conductometric method^29^. The concentration of soil organic carbon (SOC) was assessed using the wet oxidation method as described by Walkley and Black^30^. Determining dissolved organic carbon (DOC) was conducted using the dichromate acid oxidation method, as described by Ciavatta et al.^31^. The Kjeldahl-distillation method determined the available nitrogen (N), as outlined by Subbaiah and Asija^32^. The available phosphorus (P) in soil samples was assessed by extracting the soil with 0.5 M sodium bicarbonate and measuring P concentration in the extract colorimetrically at 660 nm^33^. Available S was determined by turbidimetric evaluation at 420 nm wavelength^34^.

#### Enzymes activity

The dehydrogenase activity was assessed by measuring the rate at which tri-phenyl formazan (TPF) was produced from 2,3,5-triphenyl tetrazolium chloride (TTC) as outlined by Casida et al.^35^. The activity of the β-glucosidase enzyme was estimated by determining the amount of *p*-nitrophenol produced when the soil was incubated with a buffered solution (pH 6.0) containing *p*-nitrophenyl-β-d-glucoside (PNG) and toluene, following the method described by Eivazi^36^. The urease activity was measured according to the method proposed by Douglas and Bremner^37^. The activity of Alkaline phosphatase (APA) was determined by using the substrate *p*-nitrophenyl phosphate (PNP) and measuring the absorbance of the yellow colour at 420 nm^38^.

### Statistical methods

Statistical analysis was conducted using the software STATISTICA 6.0 by Stat Soft, Inc. (2001). A box plot and principal component analysis (PCA) were conducted to evaluate the variation accounted for by the principal components (PCs) using Origin (Pro) Version^39^. The two most influential principal components, which explained the most significant amount of variability, were visually depicted in a two-dimensional graph.

## Results

The long-term effects of different seasons of FYM, levels of FYM, and fertilizer N application on soil fertility and enzyme activity at various stages of wheat growth (stage 1: sowing, stage 2: tillering, stage 3: heading, stage 4: maturity, and stage 5: post-harvest) in sandy loam soil are as follows:

### Soil reaction

**Fig. 1:**
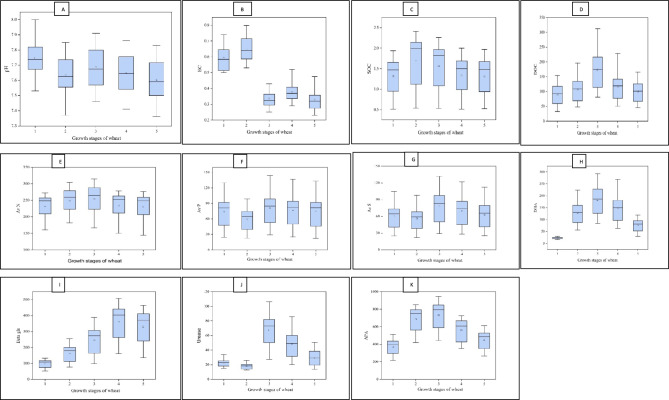
Box plot of soil pH (**A**), EC (**B**), SOC (**C**), DOC (**D**), available N (**E**), P (**F**), S (**G**), dehydrogenase activity (**H**), β-Glucosidase (**I**), urease (**J**) and alkaline phosphatase activity (**K**) at different growth stages of wheat in soils under pearl millet-wheat cropping system.

The soil pH values at stage 1, stage 2, stage 3, stage 4, and stage 5 were 7.64–7.92, 7.49–7.80, 7.54–7.87, 7.49–7.82, and 7.45–7.78, respectively (Table 1). The application methods of FYM had a substantial impact on soil pH, except for stage 5. The use of FYM resulted in the lowest soil pH at all stages of wheat growth. Applying FYM in both seasons resulted in a considerable decrease in soil pH compared to its application in *kharif* season. During stage 1, the soil pH levels between FYM_15_ and FYM_10_ treatments and between FYM_10_ and FYM_5_ treatments were statistically similar. However, beyond this stage, the soil pH values at all FYM levels showed substantial differences. The treatment of 15 t FYM ha^−1^ resulted in the most substantial decrease in soil pH compared to lesser application rates at all wheat growth stages (Table 1). Applying fertilizer N resulted in a significant decline in soil pH values at all stages evaluated, with the lowest soil pH (7.57) observed at stage 5. All three components generally exhibited a decline in soil pH from stage 1 to stage 5. The box plot revealed that during stage 1, 50% of the soil pH values fell within the range of 7.67–7.82, while during stage 2, the range was somewhat lower at 7.56–7.74. However, the lowest pH values were seen during stage 5, with 50% of the values falling between 7.50 and 7.72 (Fig. 1A). The three components did not significantly interact with soil pH in the current long-term experiment (Data not provided).

**Table 1:** Long-term impact of mode, level of FYM, and fertilizer N application on soil reaction, electrical conductivity (EC), and organic carbon at different growth stages of wheat.

Treatment	Season/level of input	Soil pH (1:2)					Soil EC (dS m^−1^)					Soil organic carbon (%)				
		Stage 1	Stage 2	Stage 3	Stage 4	Stage 5	Stage 1	Stage 2	Stage 3	Stage 4	Stage 5	Stage 1	Stage 2	Stage 3	Stage 4	Stage 5
Mode of FYM application	*Rabi*	7.79	7.64	7.69	7.65	7.62	0.57	0.64	0.32	0.37	0.34	1.29	1.70	1.55	1.31	1.30
	*Kharif*	7.78	7.67	7.72	7.69	7.64	0.58	0.62	0.32	0.35	0.28	1.23	1.54	1.45	1.26	1.22
	*Rabi* and *Kharif*: both seasons	7.68	7.61	7.65	7.61	7.56	0.63	0.68	0.36	0.41	0.37	1.43	1.81	1.69	1.46	1.45
CD (*p* = 0.05)		0.06	0.05	0.04	0.05	NS	0.05	NS	0.03	0.03	0.02	0.05	0.08	0.07	0.05	0.06
Level of FYM (t ha^−1^)	FYM_0_	7.92	7.80	7.87	7.82	7.78	0.50	0.56	0.27	0.32	0.32	0.55	0.58	0.57	0.56	0.55
	FYM_5_	7.74	7.67	7.71	7.68	7.65	0.58	0.63	0.33	0.36	0.29	1.43	1.91	1.75	1.48	1.44
	FYM_10_	7.69	7.59	7.63	7.60	7.55	0.62	0.68	0.35	0.39	0.32	1.58	2.05	1.90	1.58	1.57
	FYM_15_	7.64	7.49	7.54	7.49	7.45	0.67	0.72	0.38	0.44	0.39	1.71	2.20	2.04	1.76	1.73
CD (*p* = 0.05)		0.07	0.05	0.05	0.06	0.08	0.06	0.07	0.04	0.03	0.02	0.06	0.10	0.08	0.06	0.07
Level of fertilizer N (kg ha^−1^)	N_0_	7.78	7.67	7.72	7.68	7.64	0.57	0.62	0.31	0.35	0.31	1.27	1.64	1.52	1.30	1.28
	N_120_	7.72	7.61	7.66	7.61	7.57	0.61	0.67	0.36	0.40	0.35	1.36	1.72	1.61	1.39	1.37
CD (*p* = 0.05)		0.05	0.06	0.04	0.04	0.05	NS	0.03	0.02	0.02	0.02	0.05	0.06	0.07	0.06	0.07

The subscript figures in treatment indicate the dose of FYM in t ha^−1^ and the dose of fertilizer nitrogen in kg ha^−1^.

### Soil electrical conductivity (EC)

**Fig. 2:**
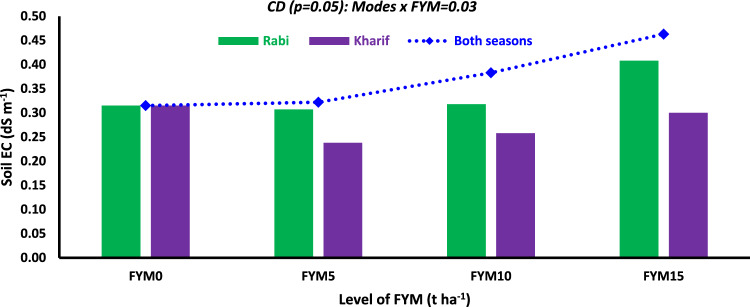
Effect of mode × level of FYM on soil electrical conductivity (EC) at stage-5 of wheat (The subscript figures in treatment indicate the dose of FYM in t ha^−1^).

The soil EC varied between 0.50 and 0.67 dS m^−1^ at stage 1, between 0.56 and 0.72 dS m^−1^ at stage 2, between 0.27 and 0.38 dS m^−1^ at stage 3, between 0.32 and 0.44 dS m^−1^ at stage 4, and between 0.28 and 0.39 dS m^−1^ at stage 5 (Table 1). The different modes, levels of FYM, and chemical N application significantly affected the soil EC. The soil EC exhibited an opposite pattern compared to the soil pH. In both seasons, the application of FYM resulted in the greatest EC values (ranging from 0.36 to 0.68 dS m^−1^), followed by the single-season application. Compared to stage 1, applying FYM decreased soil EC during *rabi*, both seasons and *kharif* season (Table 1). The application rates of FYM had substantially impacted soil EC at all stages. The EC was seen to rise as the FYM rates rose. The soil EC increased during the initial stages of wheat growth until stage 2 and then declined after crop harvest. The box plot revealed that 50% of the soil EC values ranged from 0.59 to 0.72 dS m^−1^ at stage 2, whereas at stage 5, the range was 0.27–0.36 dS m^−1^ (Fig. 1B). Regardless of the growth stages, the treatment FYM_15_ exhibited the highest soil EC, which was significantly greater than FYM_10_ at stage 4 and stage 5. During stage 1, the soil EC dropped by 36.0, 50.0, 48.4, and 41.8% under the treatments FYM_0_, FYM_5_, FYM_10_, and FYM_15_, respectively, in stage 5. Except for stage 1, applying fertilizer N substantially impacted increasing soil EC. However, the EC dropped as the growth period progressed. Additionally, applying chemical N considerably lowered soil EC at stage 5 compared to stage 1 (Table 1). At stage 5, there was a significant interaction between mode × FYM. The maximum soil EC of 0.46 dS m^−1^ was observed when 15 t FYM ha^−1^ was applied across both seasons. This was followed by the same dose of FYM supplied in the rabi season (0.41 dS m^−1^) and *kharif* season (0.30 dS m^−1^) (Fig. 2). A similar pattern was observed when applying 10 t FYM ha^−1^, however, all seasons and the *rabi* season showed no significant difference under the treatment of 5 t FYM ha^−1^. Soil EC increased with nitrogen management approaches across all three components and modes of FYM level. However, a decrease in soil EC values was seen at stage 5 compared to stage 1.

### Soil organic carbon (SOC)

Throughout 51 cycles of wheat under pearl millet-wheat farming, the SOC levels varied between 0.55 and 1.71% at stage 1, between 0.58 and 2.20% at stage 2, between 0.57 and 2.04% at stage 3, between 0.56 and 1.76% at stage 4, and between 0.55 and 1.73% at stage 5 (Table 1). The different modes of FYM application, quantities of FYM, and application of chemical N in these soils considerably influenced the SOC content. Regardless of the growth stages of wheat, application of FYM in both seasons resulted in a considerable increase in SOC, with the following order of accumulation: both seasons > *rabi* season > *kharif* season. The SOC exhibited a rise throughout the initial stages of growth until stage 2, followed by a subsequent drop. However, at stage 5, there was a slight increase in SOC compared to stage 1, except when FYM was added during the *kharif* season. The box plot indicated that at stage 2, 50% of the results for SOC were within the range of 1.12 to 2.15% (Fig. 1C). The application of 15 t FYM ha^−1^ increased SOC content as follows: stage 1: 8.2%, stage 2: 7.3%, stage 3: 7.4%, stage 4: 11.4%, and stage 5: 10.2%, compared to the application of 10 t FYM ha^−1^ (Table 1). Applying fertilizer N considerably increased the SOC content by 4.9–7.1% at various stages of the wheat growing period. At stage 5, the N_120_ treatment resulted in a higher SOC increase than the N_0_ treatment (Table 1). The long-term data showed that different combinations of modalities and quantities of FYM substantially impacted the SOC content during all growth stages of wheat (Table 2). The SOC varied between 0.55 and 1.89% at stage 1, 0.58 and 2.36% at stage 2, 0.57 and 2.22% at stage 3, 0.56 and 1.94% at stage 4, and 0.55 and 1.91% at stage 5. Except for *rabi* × FYM_15_ at stage 2, both seasons × FYM_15_ showed noticeably increased SOC content at all growth stages compared to either single season × FYM_15_. Regardless of the growth stages, the treatment of both seasons with FYM_15_ significantly enhanced the SOC compared to both seasons with FYM_10_, except for stage 2 (Table 2). No significant interactions were observed for SOC in this experiment. However, the specific data on these interactions is not provided.

**Table 2:** Impact of mode × level of FYM on soil organic carbon (SOC), available P and S at different growth stages of wheat.

Wheat growth period	Mode of FYM application	Level of FYM application (t ha^−1^)											
		FYM_0_	FYM_5_	FYM_10_	FYM_15_	FYM_0_	FYM_5_	FYM_10_	FYM_15_	FYM_0_	FYM_5_	FYM_10_	FYM_15_
		Soil OC (%)				Available P (kg ha^−1^)				Available S (kg ha^−1^ )			
Stage-1	*Rabi*	0.55	1.39	1.55	1.69	26.5	69.4	80.0	87.0	27.1	62.2	68.2	74.3
	*Kharif*	0.55	1.34	1.46	1.56	26.5	69.7	82.9	92.6	27.1	52.6	57.0	65.4
	*Rabi* and *Kharif*: both seasons	0.55	1.57	1.73	1.89	26.5	92.0	113.9	126.3	27.1	72.6	83.6	99.2
	CD (*p* = 0.05)	0.11				6.5				8.9			
Stage-2	*Rabi*	0.58	1.95	2.08	2.19	25.1	62.2	68.2	74.3	24.2	57.7	63.7	70.7
	*Kharif*	0.58	1.69	1.86	2.04	25.1	53.1	58.5	65.4	24.2	51.9	55.1	64.0
	*Rabi* and *Kharif*: both seasons	0.58	2.09	2.21	2.36	25.1	73.6	82.2	94.2	24.2	66.2	75.8	93.3
	CD (*p* = 0.05)	0.17				7.9				8.1			
Stage-3	*Rabi*	0.57	1.76	1.88	2.02	32.1	76.5	86.7	95.9	31.6	75.7	86.9	95.9
	*Kharif*	0.57	1.59	1.75	1.89	32.1	72.8	82.7	92.8	31.6	69.3	78.5	93.3
	*Rabi* and *Kharif*: both seasons	0.57	1.91	2.07	2.22	32.1	100.1	126.1	139.3	31.6	94.6	116.5	128.1
	CD (*p* = 0.05)	0.14				7.1				6.8			
Stage-4	*Rabi*	0.56	1.43	1.54	1.73	30.0	72.5	84.0	92.2	30.4	67.0	76.0	86.7
	*Kharif*	0.56	1.40	1.47	1.61	30.0	68.3	80.7	88.9	30.4	60.3	72.2	83.4
	*Rabi* and *Kharif*: both seasons	0.56	1.61	1.75	1.94	30.0	96.4	121.5	133.1	30.4	83.9	97.0	116.0
	CD (*p* = 0.05)	0.11				7.8				5.7			
Stage-5	*Rabi*	0.55	1.41	1.53	1.71	25.0	71.3	82.5	89.7	28.5	65.5	72.1	79.6
	*Kharif*	0.55	1.32	1.44	1.56	25.0	67.4	80.8	89.3	28.5	51.7	56.0	65.1
	*Rabi* and *Kharif*: both seasons	0.55	1.59	1.75	1.91	25.0	94.2	117.5	129.9	28.5	78.3	90.4	106.7
	CD (*p* = 0.05)	0.13				6.5				6.8			

The subscript figures in treatment indicate the dose of FYM in t ha^−1^.

### Soil dissolved organic carbon (DOC)

The soil's DOC levels varied at different growth stages. In stage 1, the range was from 32.5 to 154.3 mg kg^−1^. In stage 2, it ranged from 47.5 to 196.5 mg kg^−1^. In stage 3, the range was from 81.5 to 312.1 mg kg^−1^. In stage 4, it ranged from 50.6 to 228.1 mg kg^−1^. Lastly, in stage 5, the range was from 45.2 to 165.8 mg kg^−1^. The use of FYM in both seasons significantly increased the DOC content compared to applying it in a single season, regardless of the stages (Table 3). Except for stage 1, the application of FYM during the *rabi* season yielded much superior results compared to its application during the *kharif* season. A statistically significant increase in DOC from 5.9 to 20.7% was observed at stage 5 compared to stage 1 of the wheat growing period. The relative abundance of DOC varied depending on the mode of FYM application, with the highest abundance seen in stage 3, followed by stage 4, stage 2, stage 5, and stage 1. At stage 3, the box plot indicated that 50% of the values for DOC fell within the range of 113.9 to 216.2 mg kg^−1^ (Fig. 1D). The application of 15 t FYM ha^−1^ resulted in a considerable increase in DOC content at different stages of growth (stage 1: 11.2%, stage 2: 10.4%, stage 3: 10.5%, stage 4: 10.1%, and stage-5: 9.1%), compared to the application of 10 t FYM ha^−1^ (Table 3). Like SOC, adding N significantly augmented the DOC levels during various wheat growth stages. The range of mode × level of FYM varied from 41.9 to 140.9, 55.6 to 169.3, 89.6 to 268.7, 59.7 to 190.8, and 51.6 to 149.6 mg kg^−1^ at stages 1–5, respectively (Table 3). Although stage 1 had an impact, the levels of modes × level of FYM had a substantial effect on DOC content. In the other growth stages, the application of 15 t FYM ha^−1^ during both seasons was significantly better. The interaction between FYM and fertilizer N considerably impacted the DOC concentration in soil, similar to the effect observed in modes × amount of FYM (Table 3). Treatment FYM_15_ × N_120_ resulted in a significantly increased content of DOC compared to treatment FYM_10_ × N_120_. However, at stage 2 and stage 3, treatment FYM_15_ × N_120_ showed no significant difference in DOC content compared to treatment FYM_10_ × N_120_. The treatment FYM_15_ × N_120_ was determined to be more effective in increasing the DOC in the soil compared to other combinations, including the use of FYM and N fertilization (Table 3). The use of fertilizer N in mode × had a considerable impact on soil DOC during stage 3 and stage 4. In both seasons, applying fertilizer N also considerably enhanced DOC levels (Table 3). Ultimately, it was determined that applying FYM_15_ × N_120_ during both seasons is crucial for increasing the DOC concentration in these soils. Other interactions did not differ significantly for DOC content in soil (Data not given).

**Table 3:** Long-term impact of mode, level of FYM, and fertilizer N application on dissolved organic carbon (DOC: mg kg^−1^) at different growth stages of wheat.

Wheat growth period	Level of fertilizer N	Mode of FYM application															
		*Rabi*				*Rabi* × fertilizer N	*Kharif*				*Kharif* × fertilizer N	*Rabi* and *Kharif*: both seasons				Both seasons × fertilizer N	
		Level of FYM (t ha^−1^)					Level of FYM (t ha^−1^)					Level of FYM (t ha^−1^ )					Fertilizer N
		FYM_0_	FYM_5_	FYM_10_	FYM_15_		FYM_0_	FYM_5_	FYM_10_	FYM_15_		FYM_0_	FYM_5_	FYM_10_	FYM_15_		
Stage-1	N_0_	32.5	67.3	72.5	81.8	63.5	32.5	73.1	80.5	94.8	70.2	32.5	91.0	111.0	127.6	90.5	74.8
	N_120_	51.3	105.6	114.4	120.6	98.0	51.3	108.8	112.1	123.6	98.9	51.3	130.9	141.4	154.3	119.4	105.5
	Mode × Level of FYM	41.9	86.4	93.4	101.2	80.7	41.9	90.9	96.3	109.2	84.6	41.9	110.9	126.2	140.9	105.0	
	*CD (p* = *0.05): Mode* = *9.4; Level of fertilizer N* = *9.2; Mode* × *Level of FYM* = *NS; Mode* × *Level of fertilizer N* = *NS;Mode* × *Level of FYM* × *Level of fertilizer N* = *NS*																
Stage-2	N_0_	47.5	81.3	92.3	105.3	81.6	47.5	74.3	77.4	91.3	72.6	47.5	113.6	124.8	142.1	107.0	87.1
	N_120_	63.8	121.4	136.9	146.6	117.2	63.7	113.5	123.8	132.3	108.3	63.8	158.9	182.1	196.5	150.3	125.3
	Mode × Level of FYM	55.6	101.3	114.6	125.9	99.4	55.6	93.9	100.6	111.8	90.5	55.6	136.2	153.4	169.3	128.7	
	*CD (p* = *0.05): Mode* = *8.1; Level of fertilizer N* = *7.9; Mode* × *Level of FYM* = *16.1; Mode* × *Level of fertilizer N* = *NS; Mode* × *Level of FYM* × *Level of fertilizer N* = *NS*																
Stage-3	N_0_	81.5	146.6	160.3	208.6	149.2	81.5	130.1	138.0	153.3	125.7	81.5	212.6	201.4	225.3	180.2	151.7
	N_120_	97.8	186.2	219.9	235.4	184.8	97.8	162.5	187.1	194.3	160.4	97.8	244.3	295.5	312.1	237.4	194.2
	Mode × Level of FYM	89.6	166.4	190.1	222.0	167.0	89.6	146.3	162.6	173.8	143.1	89.6	228.4	248.4	268.7	208.8	
	*CD (p* = *0.05): Mode* = *7.2; Level of fertilizer N* = *7.0; Mode* × *Level of FYM* = *14.4; Mode* × *Level of fertilizer N* = *12.1; Mode* × *Level of FYM* × *Level of fertilizer N* = *NS*																
Stage-4	N_0_	50.6	93.3	104.0	113.6	90.4	50.6	85.5	90.1	101.2	81.9	50.6	123.9	140.1	153.5	117.0	96.4
	N_120_	68.8	125.4	143.6	154.3	123.0	68.8	126.6	131.4	137.9	116.2	68.8	166.1	197.9	228.1	165.2	134.8
	Mode × Level of FYM	59.7	109.3	123.8	133.9	106.7	59.7	106.1	110.7	119.6	99.0	59.7	145.0	169.0	190.8	141.1	
	*CD (p* = *0.05): Mode* = *6.1; Level of fertilizer N* = *3.7; Mode* × *Level of FYM* = *12.3; Mode* × *Level of fertilizer N* = *6.5; Mode* × *Level of FYM* × *Level of fertilizer N* = *NS*																
Stage-5	N_0_	45.2	85.6	97.8	104.3	83.2	45.2	76.8	83.5	97.6	75.8	45.2	97.6	123.8	133.5	100.0	86.3
	N_120_	57.9	119.8	126.5	142.1	111.6	57.9	111.7	118.9	125.5	103.5	57.9	142.0	153.9	165.8	129.9	115.0
	Mode × Level of FYM	51.6	102.7	112.2	123.2	97.4	51.6	94.2	101.2	111.6	89.6	51.6	119.8	138.8	149.6	115.0	
	*CD (p* = *0.05): Mode* = *5.8; Level of fertilizer N* = *4.0; Mode* × *Level of FYM* = *11.5; Mode* × *Level of fertilizer N* = *NS; Mode* × *Level of FYM* × *Level of fertilizer N* = *NS*																

The subscript figures in treatment indicate the dose of FYM in t ha^−1^ and the dose of fertilizer nitrogen in kg ha^−1^.

### Available nutrients in the soil

#### Available nitrogen (N)

The soil's available N content varied from 177.9 to 258.7, 193.9 to 277.4, 183.2 to 286.4, 171.9 to 263.2, and 167.4 to 260.1 kg ha^−1^ at stage 1, stage 2, stage 3, stage 4, and stage 5, respectively (Table 4). The different seasons of applying FYM had a noticeable effect on the amount of available N, except during stages 3 and 4. The application of FYM in both seasons resulted in the highest soil N levels (240.3–268.4 kg ha^−1^) across all growth stages, with the *rabi* and *kharif* seasons following closely behind. Applying 15 t FYM ha^−1^ resulted in a significant rise in available N compared to using 5 t FYM ha^−1^ at various wheat growth stages. Nevertheless, the application of 15 t FYM ha^−1^ did not result in a substantial increase in soil available N compared to the application of 10 t FYM ha^−1^. The application of fertilizer N_120_ resulted in an enhanced availability of N in the soil during various wheat growth stages in the semiarid soils of Haryana (Table 4). The box plot displayed that the median of available N values at stage 3 ranged from 223.1 to 287.9 kg ha^−1^ (Fig. 1E). Based on the statistical analysis, there were no significant differences in the interactions for the available N in these soils (data not given).

**Table 4:** Long-term impact of mode, level of FYM, and fertilizer N application on available nutrients (NPS: kg ha^−1^) at different growth stages of wheat.

Treatment	Season/level of input	Available N (kg ha^−1^)					Available P (kg ha^−1^)					Available S (kg ha^−1^)				
		Stage 1	Stage 2	Stage 3	Stage 4	Stage 5	Stage 1	Stage 2	Stage 3	Stage 4	Stage 5	Stage 1	Stage 2	Stage 3	Stage 4	Stage 5
Mode of FYM application	*Rabi*	228.2	245.8	249.8	230.8	227.3	65.7	57.5	72.8	69.7	67.1	58.0	54.1	72.5	65.0	61.4
	*Kharif*	229.8	241.0	244.0	229.7	225.6	67.9	50.5	70.1	67.0	65.6	50.5	48.8	68.2	61.6	50.3
	*Rabi* and *Kharif*: both seasons	240.9	262.8	268.4	243.6	240.3	89.7	68.8	99.4	95.3	91.6	70.6	64.9	92.7	81.8	76.0
CD (*p* = 0.05)		7.8	11.3	NS	NS	11.2	3.3	3.9	3.5	3.9	3.2	4.5	4.0	3.4	2.8	3.4
Level of FYM (t ha^−1^)	FYM_0_	177.9	193.9	183.2	171.9	167.4	26.5	25.1	32.1	30.0	25.0	27.1	24.2	31.6	30.4	28.5
	FYM_5_	243.9	258.7	268.3	247.8	244.1	77.0	63.0	83.1	79.1	77.6	62.5	58.6	79.8	70.4	65.2
	FYM_10_	251.3	269.4	278.4	256.0	252.7	92.3	69.6	98.5	95.4	93.6	69.6	64.9	94.0	81.7	72.8
	FYM_15_	258.7	277.4	286.4	263.2	260.1	101.9	78.0	109.3	104.7	102.9	79.6	76.0	105.8	95.4	83.8
CD (*p* = 0.05)		9.0	13.0	28.5	25.5	12.9	3.8	4.5	4.1	4.5	3.7	5.2	4.7	3.9	3.3	3.9
Level of fertilizer N (kg ha^−1^)	N_0_	222.8	238.7	241.9	222.9	218.8	71.7	56.3	77.5	74.0	71.8	57.4	53.6	74.6	66.3	60.0
	N_120_	243.1	261.1	266.2	246.5	243.3	77.1	61.6	84.0	80.6	77.8	62.1	58.2	81.0	72.7	65.2
CD (*p* = 0.05)		6.9	5.9	19.6	15.8	10.3	2.8	2.3	3.7	2.0	2.2	3.3	3.2	2.8	1.8	2.1

Note: The subscript figures in treatment indicate the dose of FYM in t ha^−1^ and the dose of fertilizer nitrogen in kg ha^−1^.

#### Available phosphorus (P)

The soil's available P levels varied between 26.5 and 101.9, 25.1 and 78.0, 32.1 and 109.3, 30.0 and 104.7, and 25.0 and 102.9 kg ha^−1^ throughout the first, second, third, fourth, and fifth stages of wheat growth, respectively (Table 4). All three parameters have a notable influence on the availability of P in soils. Regardless of the growth phases, applying FYM across both seasons resulted in the highest levels of available P and greatly increased the amount of P in the soil compared to just one season (Table 4). Nevertheless, the application of FYM during the *rabi* season was somewhat superior to the application during the *kharif* season, except for stage 1. Applying FYM during both seasons increased the availability of P compared to its application in the *rabi* season (Table 4). During the 51st cycle of wheat under pearl millet-wheat cropping, the application of FYM significantly impacted the availability of P in the soil. The order of influence was FYM_15_ > FYM_10_ > FYM_5_ > FYM_0_ (Table 4). Compared to the application of 10 t FYM ha^−1^, the application of 15 t FYM ha^−1^ significantly increased the availability of P by 10.4, 12.1, 11.0, 9.7, and 9.9% at stage 1, stage 2, stage 3, stage 4, and stage 5, respectively. The available P levels were lowest at stage 2 and highest at stage 3, with a subsequent drop up to stage 5 (Table 4). The box plot reveals that the lowest level of available P was observed at stage 2, while the highest level was at stage 3. Additionally, at stage 3, 50% of the values for available P varied from 53.0 to 98.2 kg ha^−1^ (Fig. 1F). Also, applying fertilizer N had a synergistic effect on the availability of P in the soil. It increased available P by 7.5, 9.4, 8.4, 8.9, and 8.4% at stage 1, stage 2, stage 3, stage 4, and stage 5 of wheat growth, respectively (Table 4). The interaction between the level of FYM for soil available P and mode × resulted in a range of 26.5 to 126.3, 25.1 to 94.2, 32.1 to 139.3, 30.0 to 133.1, and 25.0 to 129.9 kg ha^−1^ at stages 1–5, respectively (Table 2). The interaction between seasons and levels of FYM considerably affected the availability of P in the soil, regardless of the growth phases of wheat. During stage 2, the use of FYM in the rabi season had a considerably greater benefit compared to the use of FYM in the *kharif* season. However, the interaction impact of each respective single season with FYM was statistically similar for available P. Throughout all growth phases, in both seasons, the treatment combination of FYM_15_ was much better than the other treatments in retaining available P in these sandy loam soils (Table 2). No significant interactions were seen for the availability of P in the soil (data not given).

#### Available sulphur (S)

The soil's available S content varied from 27.1 to 79.6, 24.2 to 76.0, 31.6 to 105.8, 30.4 to 95.4, and 28.5 to 83.8 kg ha^−1^ throughout Stage 1, stage 2, stage 3, stage 4, and stage 5 of wheat growth, respectively (Table 4). All three elements of nutrient management impacted the availability of S in the soil. Irrespective of the phases, applying FYM during both seasons considerably increased the availability of S content in the soil by 20.0 to 27.9% compared to the *rabi* season. The order of effectiveness was found to be: both seasons > *rabi* season > *kharif* season (Table 4). The application of FYM had a considerable impact on the soil's available S content, with the highest effect observed in the FYM_15_ treatment, followed by FYM_10_, FYM_5_, and FYM_0_ (Table 4). Applying 10 t FYM ha^−1^ resulted in a considerable increase in available S at stage 1, Stage 2, stage 3, stage 4, and Stage 5. However, when a higher rate of 15 t FYM ha^−1^ was applied, the rise in available S was even more significant, with percentages of 14.4, 17.1, 12.6, 16.8, and 15.1% at each corresponding stage. The application of fertilizer N in wheat cultivation resulted in a considerable increase (8.2 to 9.7%) in the amount of available S in the soil compared to plots not treated with N (Table 4). The box plot indicated that at stage 3, the range of available S values spanned from 50.0 to 97.6, with 50% of the data falling within this range (Fig. 1G). The interaction between mode × level of FYM had a significant impact. The available S ranged from 27.1 to 99.2, 24.2 to 93.3, 31.6 to 128.1, 30.4 to 116.0, and 28.5 to 106.7 kg ha^−1^ during stages 1–5, respectively (Table 2). Throughout all stages of wheat growth, applying FYM resulted in the highest levels of available S in both the *rabi* and *kharif* seasons. Regardless of the growth phases, both seasons × FYM_15_ treatment combination exhibited superior performance compared to the other treatments in enhancing soil S availability (Table 2). The remaining interaction for the accessible S in the soil was not statistically significant (data not given).

### Enzyme activity in soil

#### Dehydrogenase activity (DHA)

The impact of various methods and levels of applying FYM and fertilizer N on dehydrogenase activity (DHA) in the soil varied within the ranges of 17.4 to 26.9, 64.5 to 174.6, 97.6 to 232.3, 73.8 to 202.6, and 37.7 to 100.0 µg TPF g^−1^ 24 h^−1^ at wheat growth stages 1, 2, 3, 4, and 5, respectively (Table 5). Except for stage 1, the application of FYM during both seasons had a considerable positive impact on DHA compared to its application in either the *rabi* season or the *kharif* season. The order of effectiveness of the application modes was as follows: both seasons > *rabi* season > *kharif* season. Applying FYM during the *rabi* season resulted in a notable rise in DHA levels compared to the *kharif* season at stage 2 and stage 4 (Table 5). The application of FYM positively affected DHA during the entire wheat growth period. Treatment FYM_15_ showed a considerable improvement in enhancing the production of DHA at stage 2, stage 3, and stage 4, whereas it was statistically similar to treatment FYM_10_ in all other aspects. Nevertheless, the utilization of 15 t FYM resulted in a notable rise in DHA levels. Specifically, there was a substantial increase of 19.8, 11.7, 22.0, 7.8, and 7.3% at stage 1, stage 2, stage 3, stage 4, and stage 5, respectively, compared to the FYM_10_ treatment (Table 5). Fertilizer N application was found to be crucial and significantly improved the development of wheat at various stages, regardless of growth stage. The value of DHA was more than 100% at stage 5 compared to its corresponding value at stage 1, among various treatment combinations. Thus, each of the three variables significantly enhanced and sustained DHA levels over wheat's growth phase (Table 5). The box plot indicated that the level of DHA was highest in stage 3, followed by stage 4, stage 2, stage 5, and stage 1 (Fig. 1H). The interaction between modes and level of FYM significantly affected DHA at stages 2–4. The range of DHA values were 64.5–211.0, 97.6–279.0, and 73.8–249.2 µg TPF g^−1^ 24 h^−1^, respectively (Table S1). The application of FYM during the *rabi* season had a statistically significant impact on DHA at stage 2 and stage 3. However, at stage 4, the combination of *rabi* × FYM_10–15_ was found to be superior in enhancing DHA compared to the comparable combination under *kharif* × FYM_10–15_ (Table S1). The impact of other interactions on DHA was determined to be statistically insignificant in these sandy loam soils (Data not provided).

**Table 5:** Long-term impact of mode, level of FYM, and fertilizer N application on soil enzyme activity at different growth stages of wheat.

Treatment	Season/level of input	Dehydrogenase (DHA: µg TPF g^−1^ 24 h^−1^)					β-Glucosidase activity (µg PNP g^−1^ h^−1^)					Urease activity (µg NH_4_^+^–N g^−1^ h^−1^)				
		Stage 1	Stage 2	Stage 3	Stage 4	Stage 5	Stage 1	Stage 2	Stage 3	Stage 4	Stage 5	Stage 1	Stage 2	Stage 3	Stage 4	Stage 5
Mode of FYM application	*Rabi*	21.8	122.5	171.5	142.2	73.9	92.5	159.5	244.8	360.9	325.2	21.4	17.5	66.3	45.3	28.5
	*Kharif*	22.4	112.6	159.6	125.4	70.4	96.9	153.1	221.3	332.3	305.6	22.1	17.1	63.9	43.3	26.4
	*Rabi* and *Kharif*: both seasons	24.7	149.3	212.2	172.0	81.6	104.0	178.2	272.8	387.0	360.5	24.0	19.7	71.5	55.2	33.5
*CD (p* = *0.05)*		*NS*	8.3	13.9	9.2	6.5	6.5	8.4	17.8	5.6	8.4	2.1	1.8	2.7	4.4	4.3
Level of FYM (t ha^−1^)	FYM_0_	17.4	64.5	97.6	73.8	37.7	57.7	83.8	106.3	169.2	146.1	15.1	13.0	31.8	22.8	15.2
	FYM_5_	23.0	127.8	186.7	143.9	70.8	102.5	168.8	265.0	399.5	371.6	22.7	17.6	71.5	47.4	28.5
	FYM_10_	24.5	145.7	207.9	166.0	92.8	111.4	192.8	295.3	422.6	390.0	24.4	19.9	77.8	56.1	34.2
	FYM_15_	26.9	174.6	232.3	202.6	100.0	119.5	209.0	318.7	449.0	414.0	27.7	21.9	87.9	65.5	40.0
*CD (p* = *0.05)*		2.8	9.6	16.0	10.6	7.5	7.5	9.7	20.6	6.4	9.7	2.5	2.1	3.2	5.0	5.0
Level of fertilizer N (kg ha^−1^)	N_0_	21.8	115.3	162.3	133.3	68.7	91.4	149.3	228.7	343.3	315.6	21.1	16.8	61.7	42.0	26.0
	N_120_	24.2	141.0	199.9	159.8	81.9	104.2	177.9	263.9	376.8	345.3	23.9	19.4	72.8	53.9	33.0
*CD (p* = *0.05)*		1.8	6.5	15.1	7.3	5.7	7.0	4.7	14.0	7.1	4.6	1.7	1.8	2.8	3.7	3.3

The subscript figures in treatment indicate the dose of FYM in t ha^−1^ and the dose of fertilizer nitrogen in kg ha^−1^.

#### β-Glucosidase activity

**Fig. 3:**
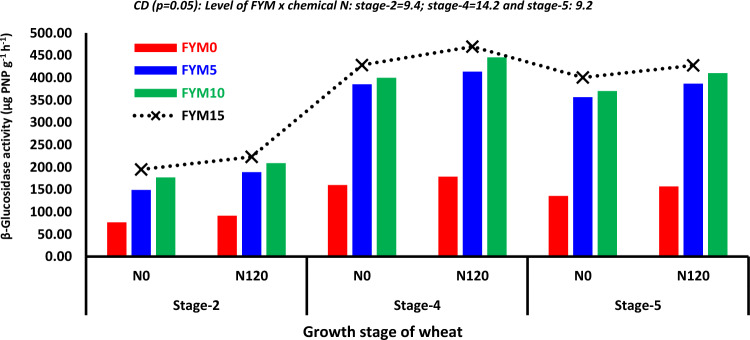
Effect of level of FYM × fertilizer N on β-Glucosidase activity at different growth stages of wheat.

The soil's β-Glucosidase activity ranged from 57.7 to 119.5, 83.8 to 209.0, 106.3 to 318.7, 169.2 to 449.0, and 146.1 to 414.0 µg PNP g^−1^ h^−1^ during the stage 1, stage 2 stage 3, stage 4, and stage 5 of wheat growth, respectively (Table 5). β-Glucosidase activity during the wheat growing phase was significantly affected by the seasons, levels of FYM, and chemical N treatment, regardless of the stages. Irrespective of the phases, both seasons of FYM application exhibited increased β-Glucosidase activity compared to the *rabi* season. The activity levels were as follows: stage-1: 12.4, stage-2: 11.7, stage-3: 11.4, stage-4: 7.2, and stage-5: 10.9% (Table 5). Applying 10 t, FYM ha^−1^ resulted in a β-Glucosidase activity equal to the higher rate only during stage 1. However, from stage 2 to stage 5, the application of 15 t FYM ha^−1^ was much more effective. The maximum β-Glucosidase activity was seen during stage 4 of the wheat growing period, regardless of FYM levels (Table 5). Fertilization with N greatly enhanced β-Glucosidase activity throughout all phases of wheat growth. Treatment N_120_ significantly increased β-Glucosidase activity at stages 1–5, with percentage increases of 14.0, 19.2, 15.4, 9.8, and 9.4% compared to treatment N_0_ (Table 5). The box plot revealed that the β-Glucosidase activity was most pronounced during stage 4 of wheat growth, in comparison to the other growth stages (Fig. 1I). The interaction effect of modes × FYM on β-Glucosidase activity varied between 83.8 and 232.6, 169.2 and 485.7, and 146.1 and 451.9 (µg PNP g^−1^ h^−1^) at stage 2, stage 4, and stage 5, respectively (Table S1). Regardless of the levels of FYM, both seasons × FYM exhibited much better performance than either single season. Additionally, *rabi* × FYM outperformed *kharif* season × FYM specifically for stage 4 to stage 5. The impact of both seasons × FYM_15_ on β-Glucosidase activity was substantially higher than all other combinations. The relationship between the levels of fertilizer N and the activity of β-Glucosidase was found to be considerable, similar to the relationship shown in modes of FYM (Fig. 3). The combination of FYM_15_ × N_120_ had a significant effect compared to other combinations at stage 2 (223.0 µg PNP g^−1^ h^−1^), stage 4 (469.6 µg PNP g^−1^ h^−1^), and stage 5 (427.5 µg PNP g^−1^ h^−1^) during the wheat growing period (Fig. 3). However, there were no significant differences seen in the interactions during the other growth phases of wheat (Data not provided).

#### Urease activity

**Fig. 4:**
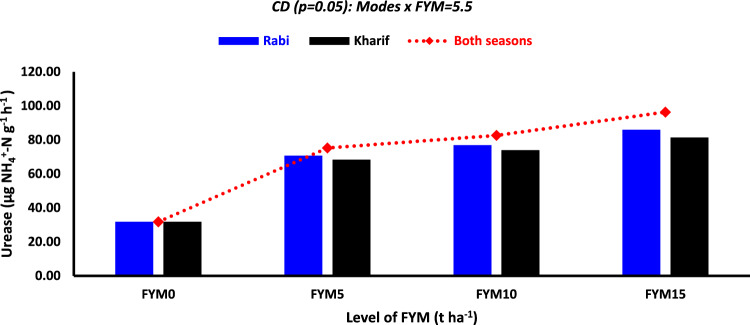
Effect of mode × level of FYM on urease activity at stage-3 of wheat growth.

The soil's urease activity varied between 15.1 to 27.7, 13.0 to 21.9, 31.8 to 87.9, 22.8 to 65.5, and 15.2 to 40.0 µg NH_4_^+^–N g^−1^ h^−1^throughout the stage 1, stage 2 stage 3, stage 4, and stage 5 of wheat growth, respectively (Table 5). Compared to each season, applying FYM considerably enhanced urease activity in all wheat growth stages, except for the *kharif *season, which performed similarly to both seasons. At stage 1, the urease activity was 22.1 µg NH_4_^+^–N g^−1^ h^−1^ for the *kharif* season and 24.0 µg NH_4_^+^–N g^−1^ h^−1^ for both seasons. The administration of FYM improved urease activity by 12.1, 12.6, 7.8, 21.9, and 17.5% at stages 1 to 5, respectively, across both seasons compared to the *rabi* season (Table 5). Regardless of the growth phases, the positive effect of FYM levels was seen, and urease activity increased as the level of FYM grew (Table 5). At stages 1–5, treatment FYM_15_ increased urease activity by 13.5, 10.1, 13.0, 16.8, and 17.0% compared to the control treatment FYM_10_. However, there was no significant difference between FYM_10_ and FYM_15_ at stage 2 and stage 5. Plots treated with fertilizer N showed a considerable increase in urease activity compared to control circumstances (N_0_). The rise in urease activity was 13.3, 15.5, 18.0, 28.3, and 26.9% at stages 1 to 5, respectively, compared to the control plots (N_0_) (Table 5). During the growing period of wheat, the order of urease activity among the phases of wheat growth was stage 3 > stage 4 > stage 5 > stage 1 > stage 2. A box plot displayed that 50% of the values for urease activity varied from 50.3 to 82.1 µg NH_4_^+^–N g^−1^ h^−1^ at stage 3 (Fig. 1J). The interaction between mode × amount of FYM was found to be significant at stage 3. The range of NH_4_^+^–N release was seen to be from 31.8 to 96.4 µg NH_4_^+^–N g^−1^ h^−1^ (Fig. 4). No substantial impact on urease activity was seen in any other interactions (Data not provided).

#### Alkaline phosphatase activity (APA)

**Fig. 5:**
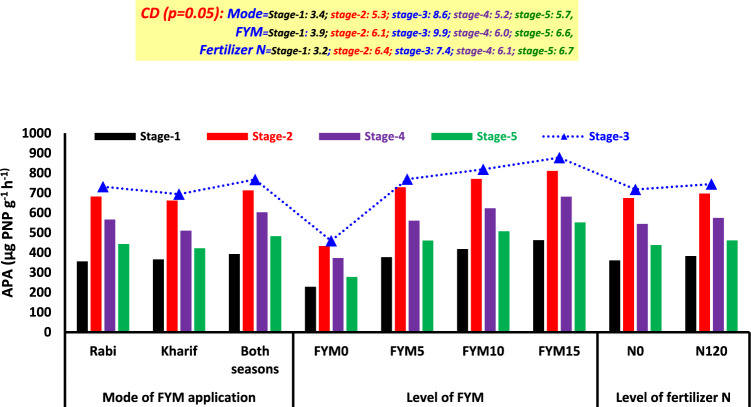
Long-term impact of mode, level of FYM and fertilizer N application on alkaline phosphatase activity (APA) at different growth stages of wheat.

**Fig. 6:**
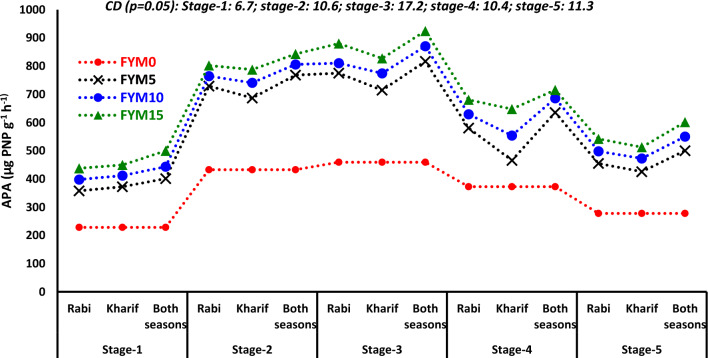
Impact of mode × level of FYM on alkaline phosphatase activity (APA) at different growth stages of wheat.

At different stages of wheat growth (stage 1, stage 2, stage 3, stage 4, and stage 5), the APA values varied from 228.5 to 462.3, 432.7 to 810.9, 459.4 to 877.3, 372.8 to 681.2, and 278.1 to 552.0 µg PNP g^−1^ h^−1^, respectively (Fig. 5). The application methods of FYM had a substantial impact on the APA similar to the available P in the soil. During various stages, the APA showed a rise of 4.4 to 10.6% when both seasons FYM were applied during the *rabi* season. However, it declined at stage 2 and then consistently increased up to stage 5 (Fig. 5). At stage 3 of wheat growth, the APA reached its peak. A box plot analysis revealed that 50% of the values ranged from 590.8 to 848.1 µg PNP g^−1^ h^−1^ at this specific growth stage (Fig. 1K). Irrespective of the phases, every level of FYM positively impacted APA. The application of 15 t FYM ha^−1^ was found to be more effective in enhancing APA than lower dosages. The application of fertilizer N had a significant impact on APA at various phases of the wheat crop (Fig. 5). Out of all the interactive effects, only the combination of mode × amount of FYM positively impacted APA at all stages. The APA values ranged from 228.5 to 499.6, 432.7 to 843.0, 459.4 to 924.4, 372.8 to 715.5, and 278.1 to 601.5 µg PNP g^−1^ h^−1^ at stages 1 to 5, respectively (Fig. 6). A notable influence of various levels of FYM on APA was observed in the following order: both seasons and level of FYM had the most significant impact, followed by the *rabi* season and level of FYM, and finally the *kharif* season and level of FYM. However, the trend for a single season was inverted for stage 1 (Fig. 6). There was no significant effect on APA from any other interactions independent of the growth phases of wheat (data not provided).

## Discussions

### Soil reaction

Soil pH is a crucial soil characteristic that provides a comprehensive understanding of the environment for plant growth, including nutrient availability, behavior of additional fertilizers, salinity/sodicity levels, soil aeration, soil mineral composition, and the prevailing meteorological conditions of the region^40^. Applying FYM for 51 years in the pearl millet-wheat cropping cycle resulted in a drop in soil pH relative to its initial value of 8.2. The use of FYM resulted in the lowest soil pH at all stages of wheat over both seasons, as shown in Table 1. The application of 15 t FYM ha^−1^ resulted in the most considerable decrease in soil pH compared to lesser FYM rates, regardless of wheat growth phases. Multiple studies have documented a reduction in soil pH following organic manures^41^. Applying fertilizer N resulted in a significant decrease in soil pH values at all stages of wheat growth. The lowest soil pH (7.57) was observed at stage 5. The trend indicated that soil pH was first at its greatest during stage 1 and then declined from stage 3 to stage 5 (Fig. 1A). Regardless of the types and amounts of organic fertilizer used, soil pH dropped during the growth phases of the wheat crop compared to the time of sowing. This decline could be attributed to the oxidation of ammonium to nitrate that occurs during the growth of crops. Humic compounds include many functional groups, such as carboxylic acids and phenolics, within their organic matrix. The drop in soil pH can be attributed to specific properties such as pH buffering, regulation of equilibrium in ion exchange, and oxidation–reduction processes^42^. The prolonged utilization of chemical fertilizers, specifically urea, as an N source has led to soil acidification^43^. This is because these fertilizers provide N in the form of NH_4_^+^, which, when oxidized, releases H^+^ ions into the soil. Over 33 years, the continuous use of FYM and mineral fertilizer in rain-fed soybean-wheat cropping systems resulted in a considerable drop in soil pH^44^. Meena et al.^45^ found that applying FYM and sewage sludge to sandy loam soil under irrigated circumstances in a pearl millet-wheat cropping system decreased soil pH. Sharma and Subehia^46^ observed a positive effect on soil pH in the rice–wheat system after 20 years of using organic and inorganic sources.

### Electrical conductivity (EC)

The soil EC was significantly affected by different modes, levels of FYM, and fertilizer N application. Regardless of the growth stages, both seasons and FYM_15_ treatment exhibited the maximum soil EC independently. The concentration of soluble salts in water extract (soil EC) is directly proportional to the amount of organic manure applied^47,48^. In comparison to stage 1, the application of FYM resulted in a decrease in soil EC by 40.4%, 41.3%, and 51.7% at stage 5, with *rabi*, both seasons and Kharif season, respectively (Table 1). The breakdown of organic manures results in the release of organic acids/compounds that react with sparingly soluble salts already present in the soil. This process either converts them into soluble salts or increases the solubility of salts^49^. Relative to stage 1, soil EC dropped 36.0% to 50.0% at stage 5 when varied amounts of FYM were applied. Except for stage 1, the application of fertilizer N significantly increased the soil EC. However, the EC dropped as the growth period progressed. Additionally, the use of chemical N lowered the EC by 42.6% at stage 5 compared to stage 1 (Table 1). The soil EC showed periodic fluctuations, with the maximum recorded at stage 2 and a considerable drop observed afterwards (Fig. 1B). The rise in EC may be attributed to the introduction of soluble salts through the addition of FYM and the presence of soluble salts in N fertilizer. The irregular pattern observed may be attributed to the mineralization of FYM during the initial phases of crop growth, which led to an increase in soil EC. Additionally, the re-adsorption of cations and anions on the soil surface during later crop growth stages could have contributed to this irregular trend^50^. Applying FYM and sewage sludge at higher rates resulted in a considerable increase in soil EC in sandy loam soil's irrigated pearl millet-mustard cropping system^45^. According to Escobar and Hue^50^, introducing organic materials into andisols increased soil EC; conversely, EC in oxisols and ultisols initially increased during the incubation period but declined subsequently. Soil EC increased with nitrogen management strategies. However, a decrease in soil EC was observed at stage 5 compared to stage 1 of wheat. The soil EC values at stage 5 were significantly lower than the safe limit (0.80 dS m^−1^) for crop development in sandy loam soils of Haryana.

### Soil organic carbon (SOC)

**Fig. 7:**
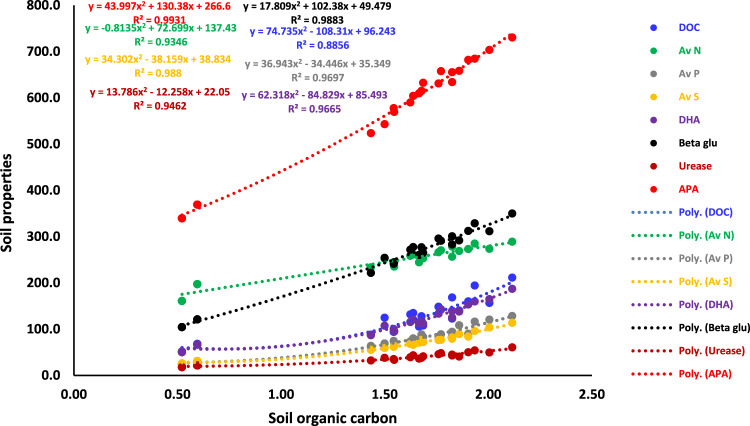
Relationship between soil organic carbon (SOC) and dissolved OC (DOC), available N, P, S, dehydrogenase activity (DHA), β-Glucosidase (Beta glu), urease and alkaline phosphatase activity (APA) of soil.

The SOC is an essential characteristic of soil health which acts as a soil conditioner, provides nutrients, serves as a substrate for microbial activity, protects the environment, and maintains crop yield^22^. The incorporation of organic materials may have increased microbial activity in the soil, leading to the formation of water-stable macro-aggregates. These aggregates are primarily held together by fungal hyphae, fibrous roots, and polysaccharides, as described by Kumari et al.^1^. The FYM application in both seasons showed greater effectiveness than either single season, resulting in a 6.5 to 11.5% improvement in SOC compared to *rabi* season (Table 1). The application of 15 t FYM ha^−1^ resulted in considerably higher SOC content than 10 t FYM ha^−1^ for the entire wheat growth period. Applying fertilizer N also resulted in a considerable increase in SOC content, ranging from 4.9 to 7.1% at various wheat growth stages. Specifically, at stage 5, treatment N_120_ was found to raise SOC by 6.9% compared to treatment N_0_, as shown in Table 1. The reason for this could be the increased crop productivity resulting from applying nitrogen fertilizers, which leads to a higher deposit of SOC^51^. The SOC increased until stage 2 and then consistently reduced. However, it was somewhat higher at stage 5 compared to stage 1 (Fig. 1C). The rise in SOC content from sowing to tillering could be attributed to the decomposition of carbon compounds facilitated by FYM application. The decline in SOC content from the growth stage to maturity could be attributed to the enhanced consumption of carbonaceous compounds by a larger population of microorganisms and higher organic matter losses as carbon dioxide (CO_2_). The pattern of OC content showed an initial increase followed by a decline toward maturity, which aligns with the observations made by Islam and Borthakur^24^. The long-term data showed that different amounts of FYM substantially impacted SOC content at all phases of wheat growth (Table 2). Overall, both seasons × FYM_15_ showed significantly greater ability to sustain the greatest level of SOC over the wheat growing period as shown in Table 2. Applying organic manures consistently, either alone or in combination with fertilizers, led to higher SOC levels than inorganic fertilizers alone, as demonstrated by studies conducted by Yaduvanshi et al.^52^. In a study conducted by Zhang et al.^53^, it was found that continuous fertilizing over 26 years increased SOC, ranging from 19.2 to 58.2% compared to the original value. The highest increase was observed in plots treated with NPK fertilizer and organic manure. The use of organic manures in combination with fertilizers has consistently resulted in the most significant OC content, as documented by several researchers^27^. Lemanowicz et al.^54^ found that the highest SOC content, measuring 8.22 g kg^−1^, was obtained while applying 60 Mg ha^−1^ of FYM. However, increasing the FYM application rate led to a 4% decrease in SOC content. The study also found a polynomial correlation between SOC and soil characteristics with an R^2^ value ranging from 0.89 to 0.99 (Fig. 7).

### Dissolved organic carbon (DOC)

Chemical extraction techniques have traditionally been used to measure labile carbon pools, which are considered early and sensitive indicators of management-induced changes in the quality and composition of SOM^11,55^. Applying FYM in both seasons resulted in a considerable increase in DOC content compared to applying it in a single season, regardless of the stages (Table 3). There was a significant increase in DOC from 5.9 to 20.7% at stage 5 compared to stage 1. The relative abundance of DOC was measured during the growing period of wheat at stage 3, as shown in Fig. 1D. The inclusion of FYM may have enhanced microbial growth due to an ample supply of nutrients, hence expediting the decomposition of organic matter and elevating the concentration of DOC^56^. The application of 15 t FYM ha^−1^ resulted in a considerable increase in DOC content compared to the application of 10 t FYM ha^−1^ (Table 3). Like SOC, adding nitrogen fertilization considerably raised the DOC content from 28.0 to 43.9% during different growth stages of wheat. Except for stage 1, the various modes and levels of FYM significantly affected DOC content. Applying 15 t FYM ha^−1^ in both seasons was the most effective combination for raising the DOC content in semi-arid soils during subsequent growth stages. The combination of treatment FYM and chemical N substantially interacted with DOC content in the soil, as shown in Table 3. Applying FYM_15_ xN_120_ during both seasons was crucial for increasing the DOC content in these soils. Applying inorganic fertilizers and organic manures has a considerable impact on the concentration of DOC in soil^55^. The treatment of the combination of NPK and FYM at a rate of 10 t ha^−1^ year^−1^ to a sandy loam soil under a maize-wheat cropping system for 34 years resulted in a considerable increase in DOC compared to soils treated with only N, NP, or NPK^57^. There has been a notable rise in DOC levels when N is applied compared to the control. Using compost alone or in combination with N for 22 years significantly raised DOC levels in soils under a maize-wheat cropping system^58^. Banger et al.^59^ found that applying inorganic fertilizers and organic manure to sandy loam soil for 16 years caused significant changes in DOC compared to SOC in a rice-cowpea farming system. Escobar and Hue^50^ found that the concentration of soluble organic carbon reduced exponentially with time due to soil microorganisms' conversion of organic carbon to carbon dioxide. Bastida et al.^60^ also noted a rapid decline in DOC initially attributed to enhanced mineralization. The further drop in DOC at later stages may be attributed to the oxidation of soluble carbon as CO_2_ and its crop consumption. The study also found a polynomial correlation between SOC and DOC concentration, with an R^2^ value of 0.89 (Fig. 7).

### Nutrient availability in soil

#### Available N

The numerical value of available N increased till stage 3. Statistically, applying FYM during both seasons was more effective than applying it during a single season at stage 5 of wheat. However, the different FYM application methods did not maintain N availability in the soil at Stage 1 and Stage 5 (Table 4). This phenomenon could be explained by the leaching and volatilization loss of N under prevailing semi-arid circumstances in addition to the plant's usage^61^. Adding organic manure has improved the soil's physical, chemical, and microbiological characteristics, increasing the availability of N in soil^62^. The application of 15 t FYM ha^−1^ resulted in a substantial increase of 6.1 to 7.2% in N availability compared to the application of 5 t FYM ha^−1^. However, similar to SOC and DOC content, the application of 15 t FYM ha^−1^ did not result in a substantial increase in soil available N compared to the treatment of 10 t FYM ha^−1^. Applying chemical fertilization (N_120_) increased N availability in soil from 9.1 to 11.2% during various growth stages of wheat in the soils of semiarid regions (Table 4). Hassan et al.^63^ found the highest levels of available N and P with the application of 100% of N through FYM followed by the treatment using 75% N through FYM and 25% from urea. Several research studies have found that using INM resulted in a considerable increase in available N compared to using organic manure or fertilizers alone^64^. Prakash et al.^12^ reported that the use of fertilizers, either alone or in combination with organic materials, yielded significantly better results compared to using organic manures alone. The frequent application of organic manure increased N mineralization, and the concentration of NH_4_–N released was higher with higher application rates of manures^14^. Basak and Biswas^65^ reported that applying organic manures with fertilizers showed the highest levels of mineral N, compared to using manure alone. Similar to DOC, the maximum amount of available N was observed at stage 3, followed by stage 2, stage 4, stage 5, and stage 1 (Fig. 1E). The availability of N from inorganic sources is fast but only for a limited period. On the other hand, when organic manure is applied, the availability of N is longer due to the production of complex polyphenolic compounds with high molecular weight. The early-stage increase in available N content is primarily caused by the quick hydrolysis of urea and enhanced mineralization of FYM in the presence of urea. This rise may also be attributed to the transformation of ammonium into nitrate^66^. The decrease in the amount of available N from the tillering stage to maturity may be attributed to the absorption of N by plants and the loss of N through both living and non-living processes^67^. Furthermore, the ability of free-living N-fixing microorganisms is reduced as plants mature, primarily due to a decrease in the rate at which organic matter is deposited in the rhizosphere. Furthermore, the drop in nitrogen content at later stages could be attributed to a slowed rate of N mineralization, likely caused by a decrease in organic N and accumulation of hazardous metabolites. Mineralization can also be diminished due to the decline in labile organic matter and microbial activity in later phases^53^. Akmal et al. also documented the fluctuation in N content^68^, and Srinivasan et al.^66^. Zhang et al.^53^ found that the simultaneous use of organic manure and fertilizer resulted in the maximum mineral N and NO_3_–N content levels during the tillering stage. These levels were lower at the transplanting stage and reached their lowest point during the grain-filling stage. The highest amount of N that may be obtained from organic manures is typically found at the late panicle initiation stage of the crop. This is because organic matter breaking down into minerals is slow during this time^69^. The tillering stage of the wheat crop has the highest need for N compared to the flowering and dough stages. An empirical correlation was found between the SOC and available N content of the soil, with a coefficient of determination (R^2^) of 0.93 (Fig. 7). In addition, a polynomial correlation was detected between urease activity and the available N concentration of the soil (R^2^ = 0.97; Fig. S2).

#### Available P

Applying FYM across both seasons resulted in a considerably increased amount (19.7 to 36.7%) of available P compared to applying it during a single season (Table 4). Applying organic manures, alone or in combination with N or NP fertilizers, resulted in a substantial increase in P availability in the soil^45^. Compared to the application of 10 t FYM ha^−1^, applying 15 t FYM ha^−1^ significantly increased the availability of phosphorus by 9.7% and 12.1% at different stages of growth (Table 4). The box plot demonstrates that the lowest levels of accessible P were observed during stage 2. Additionally, 50% of available P values ranged from 39.4 to 74.2 kg ha^−1^, as shown in Fig. 1F. The decrease in soil-available P during stage 2 (tillering) can be attributed to the increased need for the development of robust root systems, causing wheat plants to absorb a significant amount of P from the soil. Another potential factor could be the progressive provision of P resulting from the mineralization process of organic P derived from recently applied FYM. The decrease in P concentration following its peak could be attributed to using P by plants and the reduction in easily degradable organic phosphates^66^. The availability of P in soil is controlled by APA (alkaline phosphatase activity), and a polynomial relationship (R^2^ = 0.96) was established between APA and the amount of available P (Fig. S3). Many other workers observed that applying organic manures led to increased available P content^2,54^. Applying 10 t ha^−1^ FYM in alfisols and alluvial soils resulted in a 13–16.9% increase in available P content compared to plots without FYM or fertilizer P application^12^. The use of FYM coupled with NPK fertilizers results in the release of organically bound P and solubilization of soil P through organic acids released during the breakdown of organic matter. The long-term application of FYM also resulted in a decrease in the activity of polyvalent cations such as calcium (Ca), iron (Fe), and aluminum (Al) due to chelation leading to a reduction in P fixation^70^. However, implementing INM ensures an increase in the availability of P due to the conversion of soil pH to a neutral level, improvement in soil structure, and positive effects of organic and inorganic fertilizer inputs on soil health^11^. In addition, the application of chemical N had a synergistic effect on P availability in soil, resulting in an increase of 7.5 to 9.4% in available P over the wheat-growing period (Table 4). The FYM_15_ treatment outperformed all other treatment combinations in retaining available P in these soils for both seasons (Table 2). Conversely, Bahadur et al.^71^ found that plots receiving fertilizer had the highest levels of available P. In contrast, there was no notable difference in available P among plots treated with various types of organic manure. Singh and Prakash^72^ found that the highest accumulation of available P was achieved using INM. In another study, Akmal et al.^68^ found that the levels of available P showed a notable increase from October to November, followed by a minor fall in December and reaching their highest value in January. An observed decline in available P was noted from January to April, followed by a minor change leading up to May. A strong correlation was found between the SOC and available P content of the soil, with a coefficient of determination (R^2^ = 0.97) (Fig. 7).

#### Available S

Sulfur primarily exists in organic form as ester sulfates, which are not available to plants and need to be hydrolyzed into inorganic sulfates before plants can absorb them. Arylsulfatase is an enzyme that catalyses the cleavage of the O-S bond and plays a significant part in the process of ester sulfate mineralization in soils. The applications of FYM, either alone or in conjunction with chemical fertilizers, impact aryl sulfatase activity. Applying FYM throughout both seasons considerably increased the amount of available S in soil ranging from 20.0 to 27.9% relative to the *rabi* season (Table 4). The application of 15 t FYM ha^−1^ was much better and increased the availability of S by 12.6 to 17.1% over the wheat growth season. The utilization of FYM resulted in an augmentation of microbial biomass and enzyme activities, leading to an overall improvement in the availability of S in soil^23^. The plots treated with chemical N fertilizer exhibited a notable increase (8.2–9.7%) in the available S in the soil (Table 4). Like DOC, available N, and P, levels of available S were found to be highest at stage 3 followed by stage 4, stage 5, stage 1, and stage 2 (Fig. 1G). The lowest availability of S during stage 2 may be attributed to less mineralization caused by low temperature coupled with increased uptake by the crop. The subsequent rise in available S content may be attributed to enhanced mineralization from favorable temperatures and increased microbial activity in this semi-arid environment. Both seasons × FYM_15_ significantly improved the availability of S compared to the other treatment combinations (Table 2). Regardless of the application season, S content was shown to increase with higher rates of FYM application. However, the effect of increasing N fertilizer was not significant. The heightened level of activity may be attributed to the conducive conditions for microbes that degrade carbon, as well as an improved ability to utilize substrates^73^. Basak et al.^20^ also documented the superiority of integrated fertilization. The relationship between SOC and available S level in soil was polynomial (R^2^ = 0.99) (Fig. 7).

### Enzymes

The various stages of crop development require varying nutrient absorption, which results in fluctuating enzymatic activity in the soil^74^.

#### Dehydrogenase activity (DHA)

The soil enzyme dehydrogenase (DHA) is found in live cells, making it an indicator of the overall oxidative activity of soil microflora and a representation of microbial activity in soil^75^. When comparing the *rabi* season to the other seasons, applying FYM resulted in greater DHA levels. Specifically, in stage 1, stage 2, stage 3, stage 4, and stage 5, the DHA levels were 13.3, 21.9, 23.7, 21.0, and 10.4% higher, respectively (Table 5). Irrespective of the growth stages, the application of 15 t FYM ha^−1^ resulted in a considerable increase in DHA compared to 10 t FYM ha^−1^ treatment (Table 5). Using organic manures significantly increased DHA compared to mineral fertilizers and plots without any amendments^60^. The application of fertilizer N was crucial and considerably improved the DHA levels in wheat at various stages, increasing it from 11.0 to 23.2%. In rainfed wheat, adding NP resulted in a 22% increase in DHA compared to the control^64^. Overall, both seasons × FYM_15_ exhibited a significantly higher capacity than the other treatment combinations in maintaining soil DHA levels (Table S1). Mandal et al.^16^ previously demonstrated the highest DHA levels during the tillering stage of wheat. The maximum level of DHA in any crop is determined by the specific species of the crop being evaluated. In their study, Masto et al.^76^ discovered that the highest levels of DHA were observed during the vegetative stage of maize cropping, and these levels were sustained until the flowering stage. However, DHA levels fell during later stages of growth, which aligned with biochar application to the maize crops. Our investigation found that the abundance of DHA followed a periodic pattern, with the highest levels observed at stage 3, followed by stage 4, stage 2, stage 5, and stage 1 (Fig. 1H). This pattern of abundance is similar to the availability of nutrients. Dash et al.^77^ found that the administration of organic manure resulted in greater levels of DHA. These levels continued increasing up to 20 days of incubation but then dropped in aerobic and submerged environments. During a 60-year experiment, it was observed that the highest activity of soil DHA occurred during the heading stage of crop growth. However, after this stage, the DHA activity consistently declined. The highest DHA activity was recorded at different stages of crop growth when urea + PK + S + Zn-EDTA was applied, and the crops were covered with neem in the rice–wheat cropping system of the semi-arid region of Punjab^78^. After 47 years of long-term fertilization in humid subtropics of acidic alfisol in Ranchi under a soybean-wheat cropping system, the addition of NPK plus 0.40 t ha^−1^ lime resulted in 38.8% increase in DHA activity compared to the NPK treatment^79^. The peak activity observed at stage 3 can be due to the rise in the release of root exudates, which stimulates microbial activity. This activity gradually decreases as the crop reaches maturity^24^. The decline in maturity could be attributed to a gradual reduction in the decomposition rate caused by a lack of easily decomposable substrate towards the end of crop season^60^. The reduced DHA levels seen after harvest may be attributed to the soil's oxidation condition, which could be caused by limited moisture availability during maturation^68^. The combined use of organic manures and chemical fertilisers resulted in increased enzyme activity compared to using organic manures alone, as demonstrated by Bharti and Sharma^43^. An empirical correlation was found between the soil's SOC and DHA content, with an R^2^ value of 0.97 (Fig. 7).

#### β-Glucosidase activity

Over an extended period, the activity of β-glucosidase can indicate alterations in SOC levels. β-glucosidases are a diverse set of enzymes that participate in the hydrolysis of β-glucoside bonds in oligosaccharides and other glucoside conjugates, releasing glucose^80^. Applying FYM in both seasons resulted in a considerable increase in β-Glucosidase activity in soil at all stages, compared to using it in a single season alone (Table 5). The application of 15 t FYM ha^−1^ had a considerably more significant effect on β-Glucosidase activity than the application of 10 t FYM ha^−1^. The peak β-Glucosidase activity was at stage 4 of the wheat growth season. Furthermore, the general trend indicated a continual increase in activity up to stage 4 (Table 5). The box plot revealed that β-Glucosidase activity ranged from 262.5 to 441.0 µg PNP g^−1^ h^−1^ for 50% of the values at stage 4. In comparison, it ranged from 74.2 to 118.0 µg PNP g^−1^ h^−1^ at stage 1 (Fig. 1I). Fluctuations in enzyme activity can be caused by various variables, including changes in overall climate and local climate conditions, differences in nutrient availability, and alterations in the amount of leaf litter intake^77^. Piotrowska and Koper^81^ discovered that the activity of the β-glucosidase enzyme dramatically increased as the rate of FYM rose. However, they did not find any significant impact of N fertilization on enzyme activity, particularly at higher dosages of FYM in winter wheat crops. Adding 200 kg N per hectare boosted the levels of β-glucosidase in a long-term N fertilization experiment conducted on a wheat–soybean system^18^. The increased activity of enzymes that break down cellulose and hemicellulose is caused by the favourable conditions for microorganisms that degrade carbon, which is created by adding FYM^73^. A strong correlation (R^2^ = 0.99) was found between SOC and β-Glucosidase activity, indicating a polynomial relationship (Fig. 7). The combination of FYM_15_ × N_120_ had a considerably more significant effect than the other combinations at stage 2 and stage 5 (Fig. 3). Sharma et al.^82^ also found that the enzyme activity was maximum when FYM was used in conjunction with fertilizers, compared to using fertilizers alone.

#### Urease activity

Urease activity in soil serves as a biological indicator and is primarily emitted by plants, with a smaller contribution from soil microbes and animals^83^. Urease facilitates the more effective handling of urea fertilizers by catalyzing the hydrolysis of urea-containing fertilizers into CO_2_ and NH_3_. The soil's physical and chemical characteristics and agricultural practices affect the enzymatic activity^84^. The 15 t FYM ha^−1^ treatment resulted in a significant increase in urease activity ranging from 10.1 to 17.0% at different stages (Table 5). Introducing organic manures led to a considerable rise in urease activity^85^. The higher enzyme activity seen with the increased application rate of FYM can be related to the augmented populations of microorganisms resulting from greater carbon availability, leading to the release of additional extracellular enzymes^13^. The highest level of urease activity (49.8 µg N g^−1^ h^−1^) was observed while using a combination of 10 t FYM and 5 t compost per hectare, compared to using mineral fertilizer alone^86^. The trend of urease activity over the growing phase of wheat followed the order stage 3 > stage 4 > stage 5 > stage > stage 2 (Fig. 1J). The first decrease in enzyme activity may be attributed to the low temperature and the negative correlation between urease activity and temperature, as previously documented by Akmal et al.^68^. The increased urease activity observed in later stages can be due to higher N content, greater availability of organic matter, faster breakdown, and release of ammonium^45^. Both seasons × FYM_15_ had a considerably higher capacity for enhancing urease activity compared to the other combinations (Fig. 4). Several researchers have found that the activity of urease in soil reduces over time when urea/N fertilizers are used, relative to unfertilized soil. This is because soil microbes absorb mineral N from fertilizers^87^. In the rain-fed soybean-wheat system, the urease activity was suppressed in soils that were amended with organic material after 33 years of fertilization. The highest urease activity was recorded in unamended plots followed by plots that received NP treatments^88^. Akca and Namli^89^ found that increasing the dose of poultry litter biochar to 600 kg ha^−1^ decreased enzyme activity. Patel et al.^13^ have demonstrated that combining organic manures with mineral fertilizers leads to increased urease activity compared to using organic manures alone. Our study showed a polynomial correlation (R^2^ = 0.95) between SOC and urease activity in semi-arid soils (Fig. 7). Additionally, a correlation between urease activity and available N was also identified with an R^2^ value of 0.97 (Fig. S1).

#### Alkaline phosphatase activity (APA)

Phosphatase refers to a class of enzymes that catalyse the hydrolysis of esters and anhydrides of phosphoric acid and have a significant impact on the P cycle by liberating P. Phosphatase enzymes encompass both active and stable extracellular enzyme activity, thereby representing the maximum level of phosphatase activity in soils^90^. The higher level of APA seen in soil treated with manure and straw residues can be attributed to increased microbial activity and potentially a wider variety of phosphate-solubilizing bacteria resulting from the long-term addition of manure^91^. A notable rise in APA (10.6, 5.2, 7.2, 9.3, and 8.9% at wheat growing stages 1 to 5, respectively) was observed when treated with FYM_15_. Chemical N treatment, similar to the available P, considerably enhanced the abundance of active phosphatase enzymes in the soil (Fig. 5). Hota et al.^92^ reported more excellent alkaline phosphatase activity in NP treatment compared to the absolute control. The decrease in soil pH caused by organic acids generated during the decomposition of organic matter leads to an increase in enzyme activity. The FYM serves as a supply of C, N, and P for soil microbes, potentially enhancing the activity of APA. At stage 5, the APA was elevated by 15.4 to 24.7%, 19.4 to 22.0%, and 20.5 to 21.4% under the modes of FYM and chemical N levels, respectively, compared to stage 1 (Fig. 5). Prior studies^85^ have indicated that the application of organic manures leads to higher APA compared to chemical fertilizer and control. Additionally, APA increased with higher dosages of fertilizer. The present study found that both seasons and the degree of FYM had a significant and positive impact on APA (Fig. 6). The enzyme activity decreased with a higher dose of chicken litter biochar. However, the maximum enzyme activity was observed in the INM system compared to using organic materials alone. This finding is supported by studies conducted by Moharana et al.^64^ and Akca and Namli^89^. Our study found a strong correlation between SOC and APA with an R^2^ value of 0.99 (Fig. 7). Additionally, a high correlation was identified between APA and P with an R^2^ value of 0.96 (Fig. S2). Throughout the wheat's growth phase, the largest APA was seen during stage 3, followed by stage 2, stage 4, stage 5, and stage 1 (Fig. 1 K). The shift of soil pH towards alkaline levels increased APA due to soil alkaline conditions combined with N management measures. Seasonal fluctuations, plant developmental stage, and organic matter content are significant factors that strongly impact soil microbial biomass. DeForest et al.^93^ determined that the activity of enzymes is influenced by the initial bioavailability of P in soil. Islam and Borthakur^24^ have observed increased phosphatase activity during the robust growth stage, followed by a drop after maturity.

The soil parameters exhibited their highest values at different stages of the wheat growing period. Specifically, the highest values of soil pH, EC, SOC, DOC, available N, P, S, DHA, β-Glucosidase activity, urease, and APA were seen at stage-1, stage-2, stage-2, stage-3, stage-3, stage-3, stage-3, stage-3, stage-4, stage-3, and stage-3, respectively. Prior research has demonstrated that the activity of enzymes in wheat increased from the time of sowing to the flowering and intense growth stages, and subsequently decreased at the time of harvest^21,23^

### Principal component analysis (PCA)

**Fig. 8:**
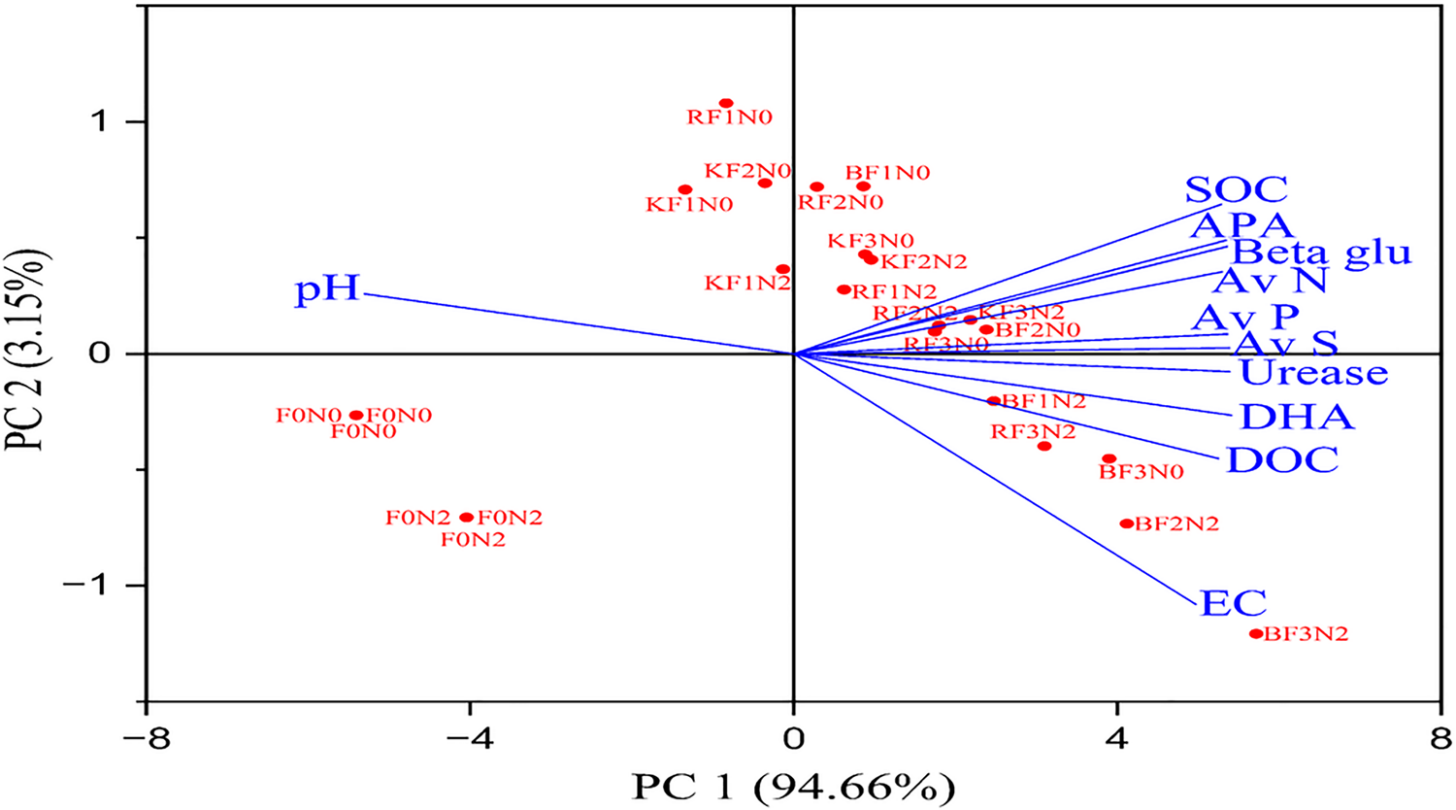
The plot of principal component analysis (PCA) on the soil properties, including soil pH, electrical conductivity (EC), soil organic carbon (SOC), dissolved OC (DOC), available N, P, S, dehydrogenase activity (DHA), β-Glucosidase (Beta glu), urease and alkaline phosphatase activity (APA) in soils (pooled data of five growth stages of wheat) under pearl millet-wheat cropping system in North-western, India. PC = principal component.

The principal component analysis (PCA) effectively distinguished the soil variables and treatments in a perpendicular space (Fig. 8). PC1 and PC2 accounted for 97.8% of the variability in the data set, with PC1 explaining 94.7% and PC2 explaining 3.15% of the overall variability. The first principal component (PC1) had an eigenvalue of 10.41, whereas the second principal component (PC2) had an eigenvalue of 0.35. Each soil variable was associated with a factor loading or eigenvector weight value, indicating its contribution to variability (Table S2). In PC1, the DHA had the highest loading value of 0.307, closely followed by available sulphur and urease activity, with a loading value of 0.306. Additionally, the DHA showed significant correlations with other markers with high loading values. In the second PC (PC2), the indicator with the highest loading value was EC (− 0.682), followed by SOC (0.406) and APA (0.308). The proportion of total variance computed based on the weight of each principal component (PC) indicates that PC1 accounts for 97% of the variance, while PC2 accounts for 3%. The PCA biplot clearly showed that applying 15 t FYM ha^−1^ along with 120 N ha^−1^ during both seasons was distinctly separated and identified as the most effective treatment compared to the other combinations in this long-term study conducted in a semi-arid location (Fig. 8).

## Conclusion

The long-term seasonal application of farmyard manure (FYM) and fertilizer nitrogen (N) substantially impacted the soil parameters. During the 51st cycle of wheat under pearl millet-wheat cropping, the impact of FYM application in different seasons on soil properties in sandy loam soils exhibited the following order: both seasons (combined treatment in both *rabi* and *kharif* seasons) had the most significant impact, followed by *rabi* season application, and then *kharif* season application. Although all three factors contributed to an increase in soil electrical conductivity (EC), the levels remained significantly below the permissible threshold of 0.80 dS m^−1^ for crop production in these sandy loam soils. The application of 15 t of FYM ha^−1^ exhibited considerably more significant levels of dissolved organic carbon (DOC), available P and S, than that of 10 t FYM ha^−1^. The application of 15 t FYM significantly increased the availability of N compared to the application of 5 t FYM ha^−1^. Irrespective of the stages, every level of FYM exhibited positive and discernable impacts on soil properties. The application of FYM_15_ enhanced DHA levels and urease activity considerably and was more effective for strengthening β-Glucosidase activity and APA activity than the FYM_10_ treatment. The application of fertilizer N significantly contributed to the accumulation of SOC and enhanced the replenishment of available nutrients (N, P, and S) in the soil solution. At various phases of wheat growth, the application of 120 kg N significantly improved DHA, β-Glucosidase, urease, and APA activity compared to no application of N. The interaction between the application of FYM_15_ in both seasons and N_120_ significantly increased the content of DOC and β-Glucosidase activity compared to other combinations. The availability of N was highly and positively correlated with urease and APA activity. The PCA biplot clearly showed that applying 15 t FYM ha^−1^ and 120 kg N ha^−1^ over both seasons was distinctly separated and was considered the most effective compared to the other treatment combinations. In PC1, the DHA had the highest loading value of 0.307, closely followed by available sulphur and urease activity, with a loading value of 0.306. Applying 15 t FYM and 120 kg N ha^−1^ proved the most effective options for improving soil quality and crop growth in sandy loam soils. It resulted in the highest organic carbon (OC) levels, increased nutrient availability, and enhanced activity of beneficial enzymes throughout the growth stages of wheat in a pearl millet-wheat cropping system.

## Supplementary Information

Supplementary Information.

## Data Availability

The datasets used and/or analyzed during the current study are available from the corresponding author upon reasonable request.
