# Supplementary material for: Long-term application of FYM and fertilizer N improve soil fertility and enzyme activity in 51st wheat cycle under pearl millet-wheat
Source: Sci Rep. 2024 Sep 17;14:21695. doi: 10.1038/s41598-024-72076-w (PMC11408662; doi:10.1038/s41598-024-72076-w)
Supplement: Supplementary file 1 — Supplementary Information. [file 41598_2024_72076_MOESM1_ESM.docx]

Manuscript ID: 7d7860e3-38cf-49fb-bbaa-500b213dd98b v2.0

**Long-term influence of FYM and fertilizer N on SOC, nutrient availability and enzyme activity in the 51^st^ wheat cycle under pearl millet-wheat system**

Sunita Sheoran^1^, Dhram Prakash^1^, Parmod Kumar Yadav^1^, and Rajeev Kumar Gupta^2,3,^*, Nadhir Al-Ansari^4,^*, Salah El-Hendawy^5^, Mohamed A. Mattar^6,^*

**Table S1.** Impact of mode x level of FYM on soil enzyme activity at different growth stages of wheat.

| Wheat growth period | Mode of FYM application | Level of FYM application (t ha^-1^) | | | | | | | |
| --- | --- | --- | --- | --- | --- | --- | --- | --- | --- |
|  |  | FYM_0_ | FYM_5_ | FYM_10_ | FYM_15_ | FYM_0_ | FYM_5_ | FYM_10_ | FYM_15_ |
|  |  | Dehydrogenase (DHA: µg TPF g^-1^ 24 h^-1^) | | | | β-Glucosidase activity (µg PNP g^-1^ h^-1^) | | | |
| Stage-1 | Rabi | 17.4 | 21.6 | 22.8 | 25.3 | 57.7 | 93.7 | 105.3 | 113.2 |
|  | Kharif | 17.4 | 22.4 | 23.5 | 26.4 | 57.7 | 101.0 | 110.4 | 118.5 |
|  | Rabi and kharif: both seasons | 17.4 | 25.1 | 27.2 | 29.0 | 57.7 | 112.8 | 118.7 | 126.9 |
|  | CD (p=0.05) | NS | | | | NS | | | |
| Stage-2 | Rabi | 64.5 | 122.0 | 140.3 | 163.4 | 83.8 | 163.6 | 187.5 | 203.2 |
|  | Kharif | 64.5 | 111.0 | 125.6 | 149.4 | 83.8 | 154.4 | 182.9 | 191.1 |
|  | Rabi and kharif: both seasons | 64.5 | 150.4 | 171.1 | 211.0 | 83.8 | 188.3 | 207.9 | 232.6 |
|  | CD (p=0.05) | 16.6 | | | | 16.9 | | | |
| Stage-3 | Rabi | 97.6 | 177.2 | 193.4 | 217.7 | 106.3 | 266.4 | 291.9 | 314.7 |
|  | Kharif | 97.6 | 161.1 | 179.6 | 200.2 | 106.3 | 229.5 | 264.4 | 284.9 |
|  | Rabi and kharif: both seasons | 97.6 | 221.7 | 250.7 | 279.0 | 106.3 | 299.0 | 329.5 | 356.4 |
|  | CD (p=0.05) | 27.7 | | | | NS | | | |
| Stage-4 | Rabi | 73.8 | 137.0 | 165.6 | 192.4 | 169.2 | 402.1 | 424.2 | 448.0 |
|  | Kharif | 73.8 | 124.2 | 137.4 | 166.1 | 169.2 | 365.4 | 381.4 | 413.2 |
|  | Rabi and kharif: both seasons | 73.8 | 170.3 | 194.9 | 249.2 | 169.2 | 430.9 | 462.4 | 485.7 |
|  | CD (p=0.05) | 18.3 | | | | 11.2 | | | |
| Stage-5 | Rabi | 37.7 | 68.9 | 92.2 | 96.8 | 146.1 | 364.3 | 381.2 | 409.1 |
|  | Kharif | 37.7 | 66.0 | 86.1 | 91.8 | 146.1 | 338.6 | 356.5 | 381.1 |
|  | Rabi and kharif: both seasons | 37.7 | 77.3 | 100.3 | 111.3 | 146.1 | 411.9 | 432.4 | 451.9 |
|  | CD (p=0.05) | NS | | | | 16.8 | | | |

Note: The subscript figures in treatment indicate the dose of FYM in t ha^-1^).

**Table S2.** Loading values and percent contribution of soil properties on the axis identified by the principal component analysis (PCA).

| Soil variables | PC1 | |  | PC2 | |
| --- | --- | --- | --- | --- | --- |
|  | Loading values | Contribution of variables (%) |  | Loading values | Contribution of variables (%) |
| pH | -0.301 | 9.08 |  | 0.163 | 3.06 |
| EC | 0.282 | 7.88 |  | -0.682 | 48.21 |
| SOC | 0.300 | 9.03 |  | 0.406 | 15.89 |
| DOC | 0.298 | 8.91 |  | -0.285 | 7.27 |
| Av N | 0.301 | 9.07 |  | 0.223 | 4.75 |
| Av P | 0.305 | 9.29 |  | 0.054 | 0.34 |
| Av S | 0.306 | 9.38 |  | 0.016 | 0.05 |
| DHA | 0.307 | 9.47 |  | -0.167 | 2.58 |
| Beta glu | 0.304 | 9.27 |  | 0.292 | 8.49 |
| Urease | 0.306 | 9.39 |  | -0.048 | 0.19 |
| APA | 0.304 | 9.22 |  | 0.308 | 9.19 |
| Eigen value |  | 10.41 |  |  | 0.35 |
| Variability (%) |  | 94.66 |  |  | 3.15 |
| Cumulative variability (%) |  | 94.66 |  |  | 97.81 |

EC: Electrical conductivity, SOC: soil organic carbon, DOC: dissolved organic carbon, Av N: available nitrogen, Av P: available phosphorus, Av S: available sulphur, DHA: dehydrogenase activity, Beta glu: beta glucosidase, APA: alkaline phosphatase activity.

**Figure S1.** Climatic conditions during the investigation period**.**

**Figure S2.** Relationship between urease activity and available N of soil.

**Figure S3.** Relationship between alkaline phosphatase activity and available P of soil.
